# Zafirlukast Induces VHL- and HIF-2α-Dependent Oxidative Cell Death in 786-O Clear Cell Renal Carcinoma Cells

**DOI:** 10.3390/ijms23073567

**Published:** 2022-03-25

**Authors:** Christopher Wolf, Sonja Smith, Sjoerd J. L. van Wijk

**Affiliations:** Institute for Experimental Cancer Research in Pediatrics, Goethe University Frankfurt, 60528 Frankfurt am Main, Germany; chriss1.wolf@web.de (C.W.); s.smith@kinderkrebsstiftung-frankfurt.de (S.S.)

**Keywords:** clear cell renal cell carcinoma, CysLTR1, Von Hippel–Landau, hypoxia-inducible factor-2α

## Abstract

Mutations in the Von Hippel–Lindau (VHL) gene are the driving force in many forms of clear cell renal cell carcinoma (ccRCC) and promote hypoxia-inducible factor (HIF)-dependent tumor proliferation, metastasis and angiogenesis. Despite the progress that has already been made, ccRCC generally remain resistant to conventional therapies and ccRCC patients suffer from metastasis and acquired resistance, highlighting the need for novel therapeutic options. Cysteinyl leukotriene receptor 1 (CysLTR1) antagonists, like zafirlukast, are administered in bronchial asthma to control eicosanoid signaling. Intriguingly, long-term use of zafirlukast decreases cancer risk and leukotriene receptor antagonists inhibit tumor growth, but the mechanisms still remain unexplored. Therefore, we aim to understand the mechanisms of zafirlukast-mediated cell death in ccRCC cells. We show that zafirlukast induces VHL-dependent and TNFα-independent non-apoptotic and non-necroptotic cell death in ccRCC cells. Cell death triggered by zafirlukast could be rescued with antioxidants and the PARP-1 inhibitor Olaparib, and additionally relies on HIF-2α. Finally, MG-132-mediated proteasome inhibition sensitized VHL wild-type cells to zafirlukast-induced cell death and inhibition of HIF-2α rescued zafirlukast- and MG-132-triggered cell death. Together, these results highlight the importance of VHL, HIF and proteasomal degradation in zafirlukast-induced oxidative cell death with potentially novel therapeutic implications for ccRCC.

## 1. Introduction

Renal cell carcinomas (RCCs) are among the most common and lethal types of genito-urinary cancers and are heterogeneous in histology, genetics and tumor behavior [1,2]. Most RCCs (~70%) can be classified as clear cell RCCs (ccRCC) and, although treatment options have improved tremendously over the last few years, ccRCC metastases still occur in around half of the patients, decreasing the 5-year survival rate to approximately 10% [2]. In addition, the incidence of RCCs is expected to further increase in the coming years [3,4]. Around 95% of ccRCC cases are sporadic and the remaining 5% of familial ccRCC cases are mostly observed in Von Hippel–Lindau disease (VHD), due to an inherited mutation of the Von Hippel–Lindau (pVHL) tumor suppressor protein on chromosome 3p [5]. VHD is a genetic disorder characterized by the formation of cysts (e.g., in the pancreas) and tumors, such as hemangioblastomas, pheochromocytoma and RCCs [5]. Bi-allelic inactivation of *VHL* or *VHL* promotor hyper-methylation and subsequent *VHL* transcriptional repression underlie two thirds of sporadic ccRCC cases, and *VHL* mutations occur at the earliest point of tumor formation [6,7]. Intriguingly, second-hit *VHL* loss-of-function (LOF) mutations are insufficient to cause ccRCC, suggesting that additional mutations are required. Indeed, recurrent mutations in *PBRM1*, *BAP1* and *TP53* and loss of copy number of *CDKN2A* and *RB1* have been described to cooperate with *VHL* inactivation in ccRCC as well [6].

The function of pVHL is well understood in the cellular responses upon the transition from normal oxygen (normoxia) to decreased concentrations of oxygen in the cellular environment (hypoxia) [5,8,9,10]. The pVHL serves as a substrate-specific adaptor in multiprotein E3 ligase complexes composed of Cul2, Elongin B and C and the RING-finger protein, Rbx1. Under normal oxygen conditions, pVHL acts as ubiquitin E3 ligase that mediates ubiquitination and proteasomal degradation of hypoxia-inducible factor (HIF)-1, -2 and -3α/β [8,9]. HIFs are transcription factors that drive the transcription of genes involved in differentiation, tumor proliferation, metastasis, angiogenesis, glycolysis, cell survival and proliferation [5,10]. In normoxia, ubiquitination of HIFs by pVHL is catalyzed by HIF hydroxylation on conserved proline residues by HIF prolyl hydroxylases (HIF-PHDs), in a reaction depending on iron, oxygen and 2-oxoglutarate [5,8,9,10]. In contrast, hypoxia (or pVHL LOF mutations observed in ccRCC) disrupt this pathway, leading to HIF-1/2α stabilization and HIF-1/2α target gene transcription that eventually trigger transcriptional adaptations in tumor metabolism, genome-wide epigenetic changes, tumor growth and metastasis [5,8,9,10]. Loss of pVHL and dysregulated HIF-1/2α strongly induce pro-survival Nuclear Factor-κB (NF-ĸB) signatures in ccRCC, leading to increased relative disease risk, dismal prognosis and reduction of overall survival through pro-inflammatory cytokine and growth factor production that stimulate oncogenesis [1]. The pVHL inhibits NF-κB signaling through repression of the CARD9/BCL10/TRAF6 complex [11], and VHL-mediated inhibition of NF-ĸB is HIF-1/2α-dependent [12,13,14]. Importantly, ccRCC is generally highly resistant to conventional therapies and approximately 50% of the patients suffer from metastatic ccRCC, with a median survival of 13 months [1,2]. The first-line treatment of metastatic ccRCC is the angiogenesis inhibitor sunitinib, a multi-tyrosine kinase inhibitor that reduces the effects deriving from several important pVHL- and HIF-dependent receptor families, such as VEGFR-1, -2 and -3 [2]. Treatment of metastatic ccRCC with sunitinib prolonged disease-free and overall survival, but resistance is acquired, leading to disease progression.

Cysteinyl leukotriene receptor 1 (CysLTR1) is a membrane-spanning G protein-coupled receptor expressed in a wide variety of cell types [15]. CysLTR1 is activated by cysteinyl leukotrienes (LTs), mainly leukotriene C4 (LTC4) and leukotriene D4 (LTD4), which are eicosanoid inflammatory mediators of lipid signaling involved in allergic and hypersensitivity reactions [15]. Ligand binding to CysLTR1 controls intracellular second messenger systems leading to activation of β-catenin, PKCα and Raf/Erk pathways and expression of genes involved in proliferation, migration and survival [16]. CysLTR1 controls vasoconstriction, bronchoconstriction and vascular permeability and therefore represents an important target for prophylactic and chronic therapy in bronchial asthma allergic rhinitis [17,18]. Importantly, leukotrienes have also been shown to affect tumor growth, angiogenesis and metastasis [19]. Intriguingly, it has been demonstrated that management of chronic asthma by long-term use of CysLTR1 antagonists, like the U.S. Food and Drug Administration (FDA)-approved zafirlukast, significantly decreases the risk of several cancer types [20]. Increased CysLTR1 expression has been reported in many forms of cancer, including RCC [21]. CysLTR1 expression is associated with poor survival of colorectal [22] and prostate cancer [23]. Importantly, zafirlukast and other leukotriene receptor antagonists inhibit tumor growth, induce apoptosis or prevent the occurrence of carcinomas in vitro and in vivo, including glioblastoma [24], lung [25] and prostate cancer [23], colon carcinoma [22,26] and triple-negative breast cancer [27]. Recently, zafirlukast has also been repurposed as inhibitor of Tumor Necrosis Factor α (TNFα)-induced TNFR1 pre-ligand assembly domain (PLAD)-mediated TNFR1 activation [28,29]. Of note, TNF Receptor 1 (TNFR1) mRNA expression has been shown to be elevated in kidney cancer, with particularly high TNFR1 expression being linked with worse outcomes in ccRCC patients [30,31].

Here, we investigate the functional mechanisms and determinants that underlie zafirlukast-mediated cell death in RCC cells. We demonstrate that zafirlukast induces a TNFα-independent mode of cell death in 786-O cells that is mechanistically unrelated to apoptosis or necroptosis. Zafirlukast-triggered cell death is highly oxidative and can be rescued by antioxidants and inhibition of Poly (ADP-ribose) polymerase 1 (PARP-1). Finally, zafirlukast-induced cell death is dependent on VHL and HIF-2α status and can be modified by inhibition of proteasomal degradation.

## 2. Results

**Figure 1:**
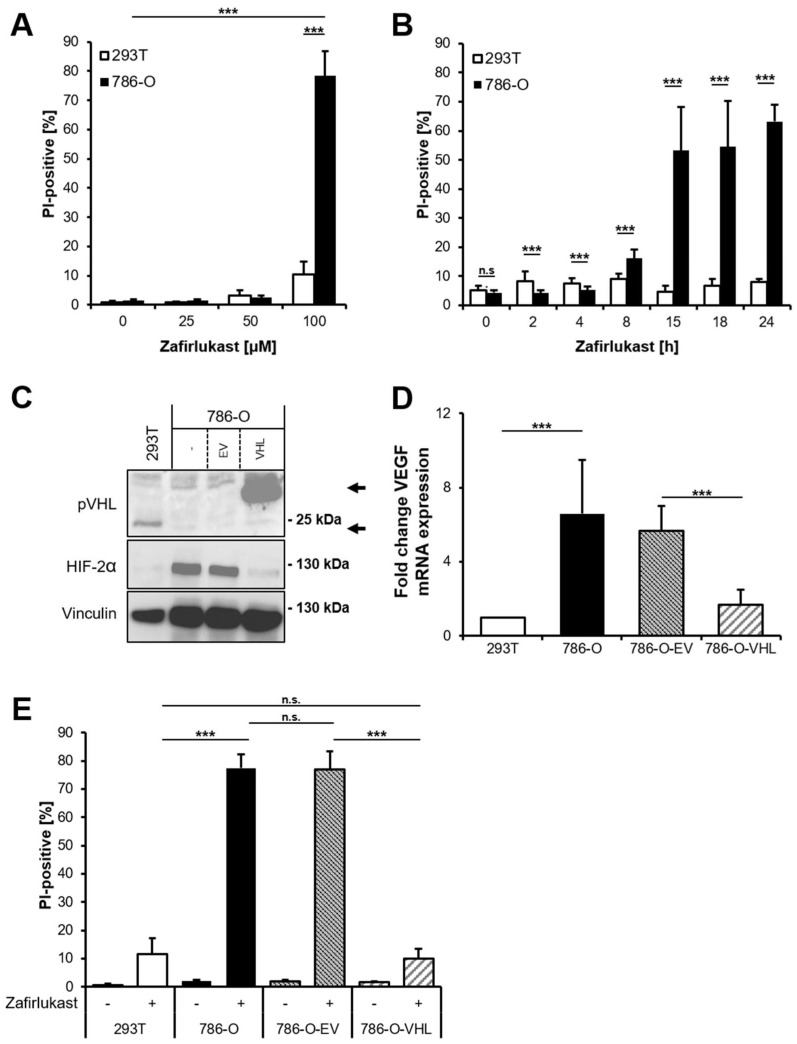
Zafirlukast triggers pVHL-dependent cell death. (**A**) 786-O and 293T cells were treated with the indicated concentrations of zafirlukast for 24 h, after which cell death was determined by quantifying PI uptake. Data are presented as percentage of PI-positive cells and mean and SD of three independent experiments performed in triplicate are shown. *** *p* < 0.001; (**B**) As in (**A**), but cells were exposed to 100 µM zafirlukast for the indicated time points. *** *p* < 0.001, n.s.: non-significant; (**C**) Western blot analysis of pVHL and HIF-2α expression in 293T, 786-O, 786-O-EV and 786-O-VHL cells. Vinculin served as loading control. Representative blots of at least three different independent experiments are shown. Lower arrow indicates endogenous pVHL, upper arrow indicates Flag-tagged reconstituted pVHL; (**D**) mRNA expression levels of *VEGF* in the indicated cell lines were determined using qRT-PCR. Data are normalized to *GAPDH* expression and are presented as x-fold mRNA expression compared to control. Mean and SD of three independent experiments performed in triplicate are shown. *** *p* < 0.001; (**E**) 293T, 786-O, 786-O-EV and 786-O-VHL were treated with DMSO or 100 μM zafirlukast for 24 h, after which cell death was determined by quantifying PI uptake. Data are presented as percentage of PI-positive cells and mean and SD of three independent experiments performed in triplicate are shown. *** *p* < 0.001, n.s.: non-significant.

The CysLTR1 inhibitor zafirlukast induces cell death in several types of tumors, but the underlying mechanisms remain largely unclear. To understand how zafirlukast controls ccRCC cell death, the *VHL*-mutated human RCC cell line 786-O was exposed to increasing zafirlukast concentrations, followed by quantification of cell death by measuring the fraction of propidium iodide (PI)-positive cells. Prominent concentration- and time-dependent cell death was induced in 786-O cells, but not in the wild-type pVHL human embryonic kidney control cell line 293T (Figure 1A,B). Additionally, 786-O cells were also more sensitive to cell death induced by the CysLTR1 inhibitor montelukast in a time- and concentration-dependent manner, compared to 293T cells (Figure S1). Since expression of pVHL is lost in 786-O cells and ccRCCs, we evaluated the mechanistic roles of functional pVHL in zafirlukast-mediated cell death. For this, *VHL*-mutated 786-O ccRCC cells were stably reconstituted with empty vector (786-O-EV) or pVHL (786-O-VHL). Western blot analysis confirmed the stable expression of FLAG-tagged pVHL in reconstituted 786-O-VHL cells, accompanied by a loss of HIF-2α, confirming the functionality of the introduced pVHL (Figure 1C). In addition, expression of pVHL was accompanied by a pVHL-dependent downregulation of the HIF-2α-induced target gene *VEGF* (Figure 1D). Surprisingly, reconstituted 786-O-VHL ccRCC cells were fully resistant against zafirlukast-induced cell death compared to 786-O-EV ccRCC cells (Figure 1E), which undergo cell death to the same extent as wild-type 786-O ccRCC cells. These findings suggest that zafirlukast triggers a pVHL- and/or HIF-2α-dependent type of cell death in ccRCC cells.

**Figure 2:**
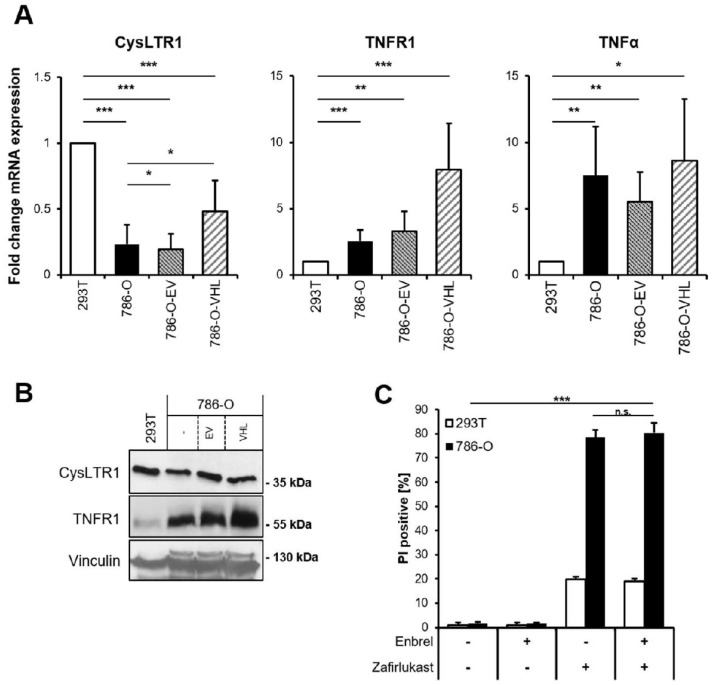
TNFα does not affect zafirlukast-induced cell death. (**A**) mRNA expression levels of *CysLTR1*, *TNFR1* and *TNFα* in the indicated cell lines were determined using qRT-PCR. Data are normalized to *GAPDH* expression and presented as x-fold mRNA expression compared to control. Mean and SD of four independent experiments performed in triplicate are shown. * *p* < 0.05, ** *p* < 0.01, *** *p* < 0.001; (**B**) Western blot analysis of CysLTR1 and TNFR1 expression in 293T, 786-O, 786-O-EV and 786-O-VHL cells. Vinculin serves as loading control. Representative blots of at least three different independent experiments are shown; (**C**) 786-O and 293T cells were pre-treated with 100 μg/mL of Etanercept (Enbrel) for 1 h, followed by treatment with DMSO or 100 μM zafirlukast for 24 h, after which cell death was determined by quantifying PI uptake. Data are presented as percentage of PI-positive cells and mean and SD of three independent experiments performed in triplicate are shown. *** *p* < 0.001, n.s.: non-significant.

Since zafirlukast might act via CysLTR1 or through TNFR1 and CysLTR1, and since TNFR1 signaling has been implicated in cell fate regulation, basal mRNA levels of *CysLTR1*, *TNFR1* and *TNFα* were analyzed. *CysLTR1* mRNA expression was shown to be lower in 786-O and 786-O-EV cells compared to 293T cells and increased upon expression of pVHL (Figure 2A). In contrast, *TNFR1* expression was discovered to be higher in 786-O cells and increased upon expression of pVHL, whereas *TNFα* expression was generally higher in 786-O cells (Figure 2A). Of note, increased TNFR1 protein expression could be detected in wild-type 786-O compared to 293T cells, and re-expression of pVHL further increased *TNFR1* expression compared to wild-type 786-O and 786-O-EV (Figure 2B). These findings might suggest a high level of constitutive NF-ĸB activation in 786-O cells that is potentially influenced by pVHL. Since TNFα signaling is known to coordinate programmed cell death pathways [32], the functional roles of TNFα-TNFR1 signaling in zafirlukast-induced cell death have been evaluated. Pretreating 786-O and 293T cells with the TNFα inhibitor Enbrel (Etanercept) prior to zafirlukast treatment did not reduce zafirlukast-mediated cell death (Figure 2C), suggesting that auto- or paracrine TNFα-mediated activation of TNFR1 is unlikely to contribute to zafirlukast-triggered cell death. The functionality of Enbrel was confirmed by inhibiting prototypic necroptotic cell death induced by TNFα, the SMAC mimetic BV6 and the pan-caspase inhibitor zVAD.fmk (TBZ) in the human colon carcinoma cell line HT-29 (Figure S2).

**Figure 3:**
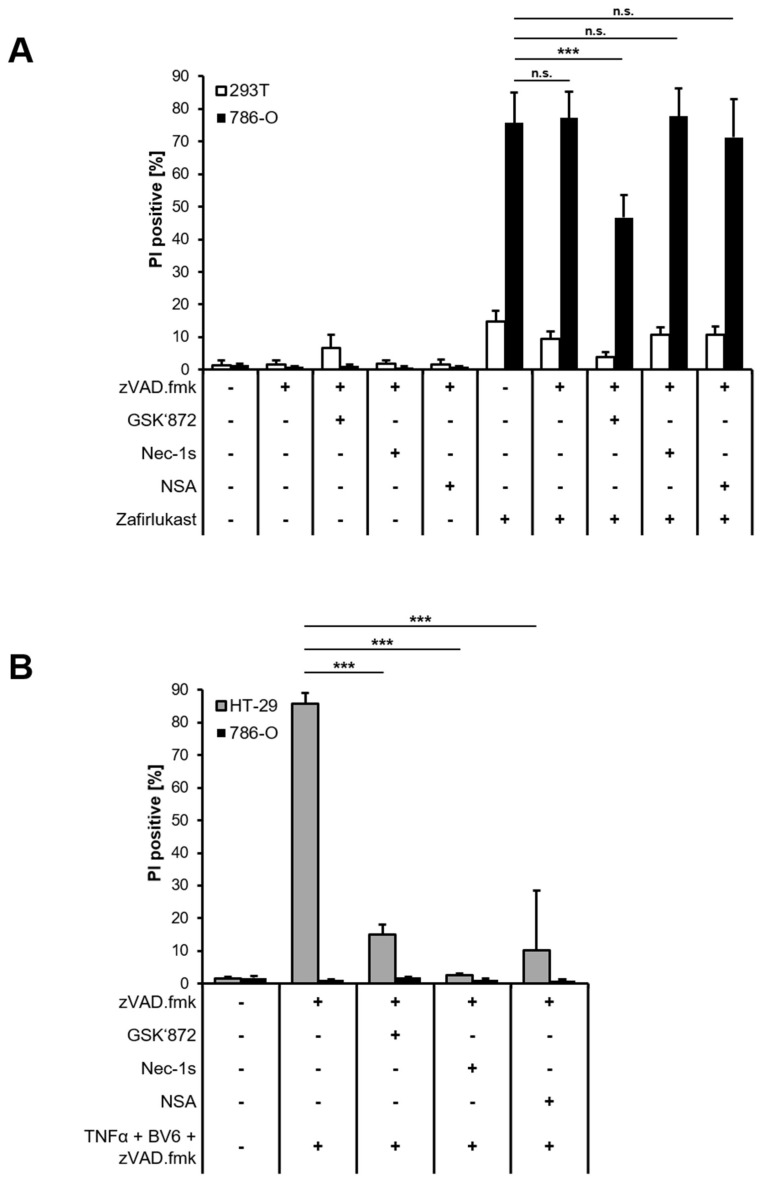
Zafirlukast does not trigger caspase-dependent apoptosis or necroptosis. (**A**) 786-O and 293T cells were pre-treated with the pan-caspase inhibitor zVAD.fmk (20 μM), the RIPK3 inhibitor GSK’872 (20 μM), the RIPK1 inhibitor Nec-1s (25 μM) or the MLKL inhibitor NSA (1 μM), followed by treatments with DMSO or 100 μM zafirlukast for 24 h, after which cell death was determined by quantifying PI uptake. Data are presented as percentage of PI-positive cells and mean and SD of three independent experiments performed in triplicate are shown. *** *p* < 0.001, n.s.: non-significant; (**B**) As in (**A**), but HT-29 and 786-O cells were treated with the indicated inhibitor/zafirlukast combinations. Data are presented as percentage of PI-positive cells and mean and SD of three independent experiments performed in triplicate are shown. *** *p* < 0.001.

To better understand the mode of cell death triggered by zafirlukast, Western blot analysis of necroptotic key proteins was performed confirming the expression of Receptor-interacting serine/threonine-protein kinase 1 (RIPK1), RIPK3, Mixed lineage kinase domain-like protein (MLKL) and caspase-8 in 786-O cells, whereas 293T cells did not express MLKL (Figure S3). Next, 293T and 786-O cells were treated with zVAD.fmk to inhibit caspase-dependent apoptosis or were exposed to zVAD.fmk combined with the RIPK1 inhibitor Necrostatin-1s (Nec-1s), the RIPK3 inhibitor GSK’872 or the MLKL inhibitor Necrosulfonamide (NSA) to inhibit necroptosis, followed by treatment with zafirlukast. Except for RIPK3 inhibition, none of the cell death inhibitors affected zafirlukast-induced cell death in 786-O cells (Figure 3A), suggesting that zafirlukast elicits neither caspase-dependent apoptosis nor necroptosis, but instead triggers a type of cell death that is at least partially dependent on RIPK3. Of note, the use of TBZ as the prototypical necroptosis stimulus in HT-29 cells confirmed the functionality of the cell death inhibitors (Figure 3B). Interestingly, despite expression of the typical necroptosis machinery, 786-O cells were unable to undergo TBZ-dependent necroptosis (Figure 3B).

**Figure 4:**
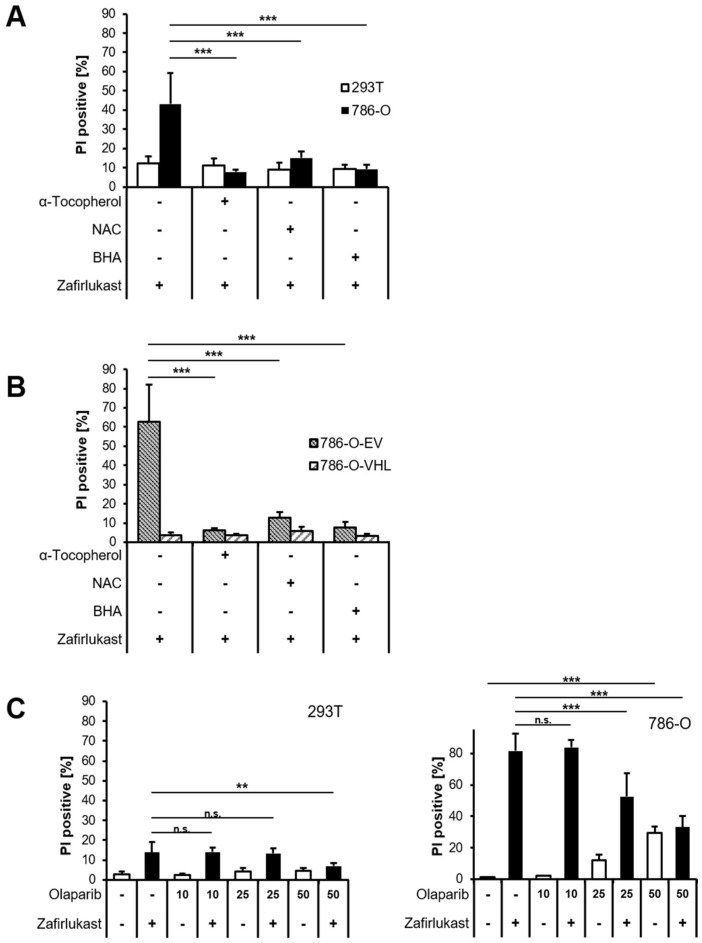
Zafirlukast elicits oxidative PARP-1-dependent cell death. (**A**) 786-O and 293T cells were pre-treated with α-Toc (100 μM), NAC (10 mM), and BHA (50 µM), followed by treatment with DMSO or 100 μM zafirlukast for 24 h, after which cell death was determined by quantifying PI uptake. Data are presented as percentage of PI-positive cells and mean and SD of four independent experiments performed in triplicate are shown. *** *p* < 0.001; (**B**) Idem as (**A**) but with 786-O-EV and 786-O-VHL cells; (**C**) 293T (**left**) and 786-O (**right**) cells were pre-treated with the indicated concentrations of the PARP-1 inhibitor Olaparib for 1 h, followed by treatment with DMSO or 100 μM zafirlukast for 24 h, after which cell death was determined by quantifying PI uptake. Data are presented as percentage of PI-positive cells and mean and SD of three independent experiments performed in triplicate are shown. ** *p* < 0.01, *** *p* < 0.001, n.s.: non-significant.

Since it appears unlikely that zafirlukast triggers caspase-dependent apoptosis or necroptosis, we hypothesized that zafirlukast-induced cell death might be associated with an increased formation of reactive oxygen species (ROS). To evaluate the role of ROS in zafirlukast-mediated cell death, 293T and 786-O cells were pretreated with α-Tocopherol (α-Toc), N-acetylcysteine (NAC), butylated hydroxyanisole (BHA), and subsequently exposed to zafirlukast. Surprisingly, zafirlukast-triggered cell death in 786-O cells could be completely rescued by α-Toc, NAC and BHA (Figure 4A), suggesting a prominent role of ROS in zafirlukast-induced cell death. As expected, zafirlukast-mediated cell death could be rescued as well in 786-O-EV cells, but did not further affect the response of 786-O-VHL cells towards zafirlukast (Figure 4B). Functionality of the antioxidants was confirmed using the ferroptosis-inducing compound erastin (Figure S4). Increased formation of ROS leads to cellular damage, including DNA single- or double-strand breaks that activate PARP-1 [33]. PARP-1 hyper-activation triggers massive poly (ADP) ribosylation, leading to energy misbalance and loss of cell viability [33]. To assess the involvement of poly ADP-ribosylation and PARP-1 in zafirlukast-induced cell death, the efficacy of the PARP-1 inhibitor, Olaparib, in zafirlukast-mediated cell death was evaluated. Indeed, Olaparib induced concentration-dependent rescue of zafirlukast-triggered cell death in 786-O, but not in 293Ts cells (Figure 4C), indicating that PARP-1 activation is involved in zafirlukast-mediated cell death.

**Figure 5:**
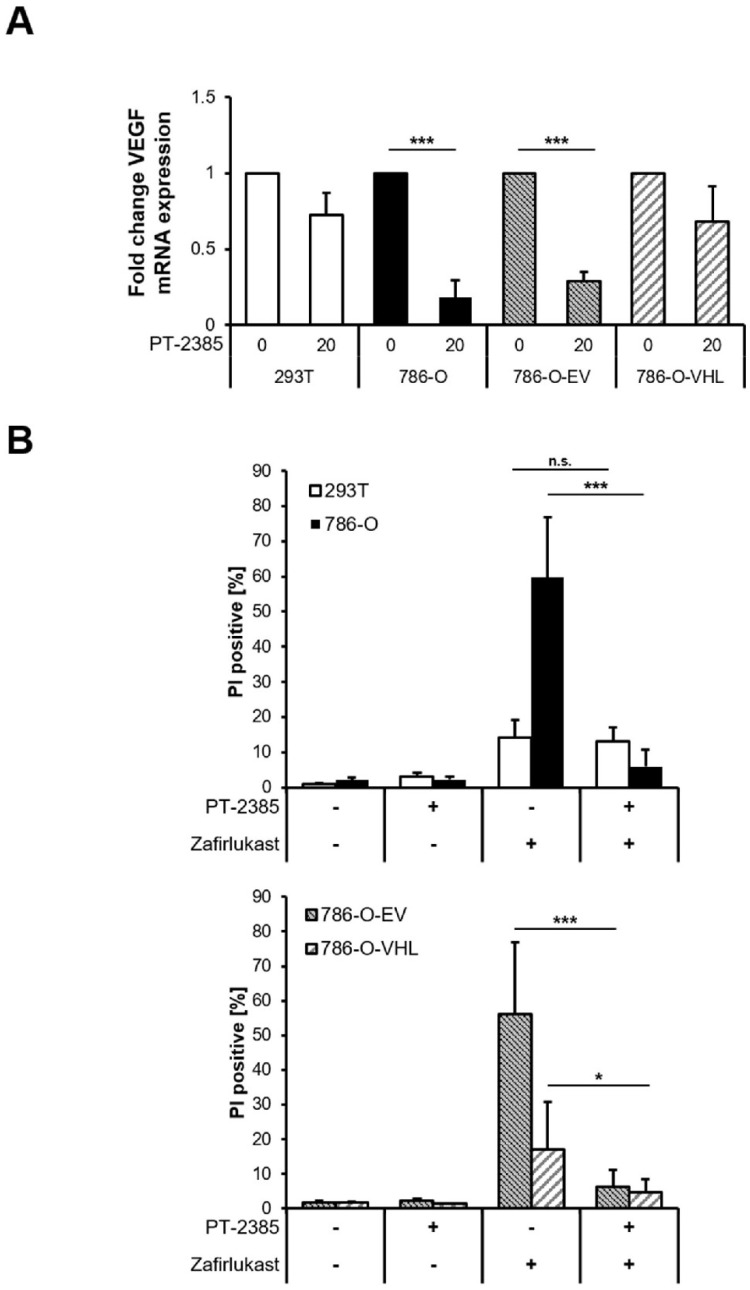
Inhibition of HIF-2α rescues zafirlukast-induced cell death. (**A**) mRNA expression levels of *VEGF* of the indicated cell lines treated with the HIF-2α inhibitor PT-2385 (20 µM) for 24 h were determined using qRT-PCR. Data are normalized to *GAPDH* expression and are presented as x-fold mRNA expression compared to control. Mean and SD of four independent experiments performed in triplicate are shown. *** *p* < 0.001; (**B**) 786-O and 293T (**upper**) and 786-O-EV and 786-O-VHL (**lower**) cells were pre-treated with the HIF-2α inhibitor PT-2385 (20 µM) for 24 h, followed by treatment with DMSO or 100 μM zafirlukast for 24 h, after which cell death was determined by quantifying PI uptake. Data are presented as percentage of PI-positive cells and mean and SD of three independent experiments performed in triplicate are shown. * *p* < 0.05, *** *p* < 0.001, n.s.: non-significant.

Loss of the tumor-suppressor pVHL is accompanied by a stabilization of HIF transcription factors that induce the upregulation of HIF-target genes responsible for ccRCC pathophysiology and carcinogenesis [10]. To understand the functional role of HIF transcription factors in zafirlukast-mediated cell death, the effects of the HIF-2α-specific inhibitor PT-2385 on zafirlukast-induced cell death were investigated. Treatment with PT2385 inhibited HIF-2α, as revealed by a pVHL-dependent downregulation of the HIF target gene *VEGF* in 786-O and 786-O-EV cells (Figure 5A). As anticipated, PT2385 did not affect *VEGF* mRNA levels in 293T and 786-O-VHL cells, due to the presence of functional pVHL (Figure 5A). To understand if HIF-2α inhibition affects zafirlukast-induced cell death, 293T and 786-O cell lines were pre-treated with PT-2385, followed by exposure to zafirlukast and quantification of cell death. Intriguingly, zafirlukast-mediated cell death could be completely reversed by PT-2385-mediated HIF-2α inhibition (Figure 5B). In addition, inhibition of HIF-2α also affected zafirlukast-induced cell death in 786-O cells reconstituted with empty vector and, to a lesser extent, also in 786-O-VHL cell lines (Figure 5B), suggesting that HIF-2α is an important mediator of zafirlukast-triggered cell death.

**Figure 6:**
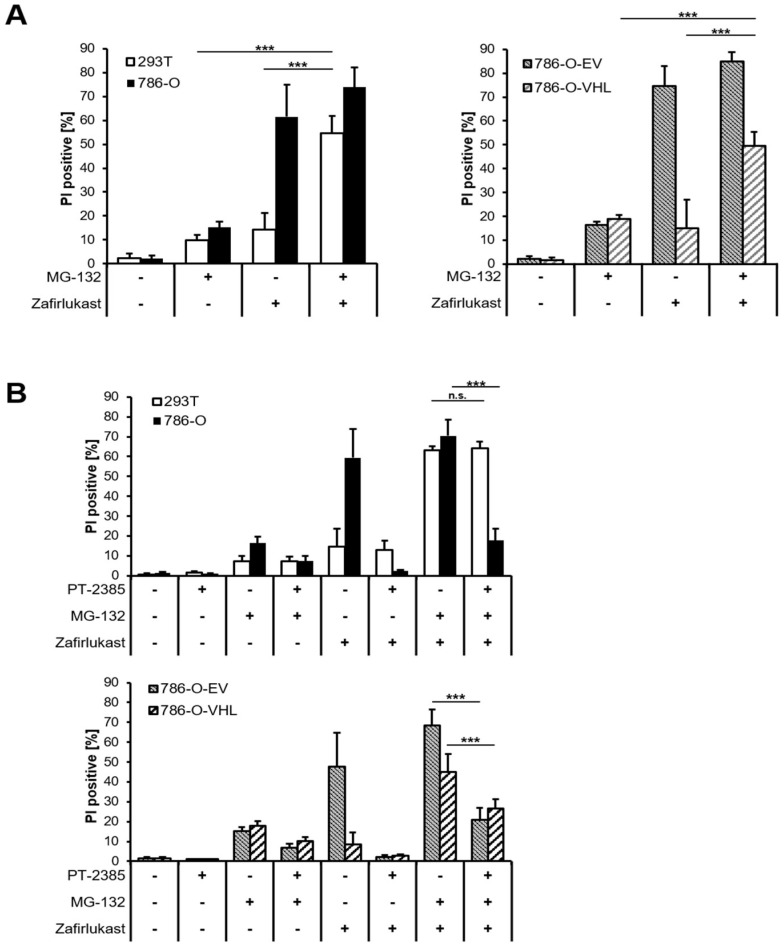
Proteasome inhibition regulates pVHL- and HIF-2α-dependent cell death triggered by zafirlukast. (**A**) 786-O and 293T (**left**) and 786-O-EV and 786-O-VHL (**right**) cells were pre-treated with the proteasome inhibitor MG-132 (10 µM) for 1 h, followed by treatment with DMSO or 100 μM zafirlukast for 24 h, after which cell death was determined by quantifying PI uptake. Data are presented as percentage of PI-positive cells and mean and SD of three independent experiments performed in triplicate are shown. *** *p* < 0.001, n.s.: non-significant; (**B**) 786-O and 293T (upper) and 786-O-EV and 786-O-VHL (lower) cells were pre-treated with the HIF-2α inhibitor PT-2385 (20 µM) for 24 h, followed by incubation with the proteasome inhibitor MG-132 (10 µM) for 1 h and treatment with DMSO or 100 μM zafirlukast for 24 h, after which cell death was determined by quantifying PI uptake. Data are presented as percentage of PI-positive cells and mean and SD of three independent experiments performed in triplicate are shown. *** *p* < 0.001, n.s.: non-significant.

The abundance of HIF transcription factors is tightly controlled by pVHL-mediated ubiquitination and degradation by the 26S proteasome. Therefore, inhibition of the 26S proteasome should sensitize wild-type pVHL-expressing cell lines towards zafirlukast-induced cell death. Indeed, treatment with the proteasome inhibitor MG-132 significantly increased zafirlukast-mediated cell death, mostly in 239T and 786-O-VHL cell lines, due to stabilization of HIF-2α degradation (Figure 6A). To confirm that MG-132- and zafirlukast-induced cell death do indeed depend on HIF-2α, cells were pre-treated with both MG-132 and PT-2385 prior to the addition of zafirlukast. Intriguingly, HIF-2α inhibition in MG-132-treated 786-O and 786-O-EV cell lines strongly reduced cell death (Figure 6B), confirming a central role of pVHL-mediated proteasomal HIF-2α degradation in zafirlukast-prompted cell death.

Together, our findings suggest that zafirlukast triggers oxidative-, caspase- and necroptosis-independent cell death, which relies on the stabilization of HIF-2α as a result of the loss of functional VHL.

## 3. Discussion

Current therapeutic interventions against ccRCC include treatment with tyrosine kinase inhibitors (e.g., sunitinib or cabozantinib), combined immunotherapy (e.g., nivolumab and ipilimumab) and targeted therapies (such as the anti-VEGF antibody, bevacizumab, or the mTOR inhibitor, everolimus) [1,34]. Although the 5-year survival rate of local stage renal cancer has improved to over 90% in the US, metastatic renal cancer still causes many deaths [35]. Therefore, there is an urgent need for novel therapeutic options to further enhance the survival of ccRCC patients.

In the present study, we have investigated the potential link between sensitivity of zafirlukast-induced cell death and pVHL status. Mutated *VHL*, as often observed in ccRCC, leads to stabilization of HIF-1/2α that activates target genes underlying ccRCC-associated alterations in tumor metabolism, genome-wide epigenetic changes, tumor growth and metastasis [5,8,9,10]. Zafirlukast is currently used as a CysLTR1 antagonist in the treatment of chronic asthma and affects leukotriene-mediated eicosanoid inflammatory signaling [17]. By doing so, zafirlukast affects cellular proliferation, migration and survival. Furthermore, leukotrienes have also been implicated in cancer cell growth, increased angiogenesis and metastasis [16]. Long-term zafirlukast treatment reduces cancer risks in cohorts of asthma patients [20] and CysLTR1 antagonists have been shown to control tumor growth and cell death in a variety of tumor entities [22,23,24,25,26,27]. In addition, zafirlukast has recently been repurposed as TNFR1 inhibitor, by interfering with ligand-independent and -dependent TNFR1 clustering [28,29].

Here, we demonstrate that zafirlukast triggers cell death in the pVHL-deficient ccRCC cell line 786-O in a time- and concentration-dependent manner, but not in pVHL-proficient 293T cells or 786-O cells that stably express a functional pVHL. Despite the upregulation of *TNFR1* and *TNFα*, zafirlukast-induced cell death could not be rescued by the TNFα-neutralizing antibody Enbrel, confirming TNFα-TNFR1-independent modes of zafirlukast-mediated cell death. TNFα is a highly pleiotropic cytokine with central roles in cell fate regulation [32]. The lack of functional TNFα-TNFR1 involvement in zafirlukast-induced cell death is of particular interest, since zafirlukast has been identified as an inhibitor of PLAD-mediated TNFR1 activation [28,29] and of TNFα-triggered inflammation in endothelial cells [36].

Zafirlukast has been described to cause cell death in many types of tumor cells [22,23,24,25,26,27] and the majority of reports link zafirlukast to triggering apoptosis, for example by inducing BCL-2-dependent apoptosis in glioblastoma cell lines [24]. However, in 786-O ccRCC cells, zafirlukast-induced cell death could not be inhibited by the caspase inhibitor zVAD.fmk, thereby excluding caspase-dependent apoptosis and suggesting highly cell- and context-specific mechanisms of zafirlukast. Although 786-O cells express caspase-8, RIPK1, RIPK3 and MLKL, zafirlukast-induced cell death could not be inhibited by common necroptosis inhibitors, ruling out necroptotic cell death as well. Interestingly, ROS scavengers rescued zafirlukast-induced cell death, suggesting an important role for ROS production and oxidative damage. One important characteristic of increased ROS production is the appearance of oxidative damage to proteins, lipids and DNA. Excessive ROS-induced DNA damage, mostly in the form of single-strand breaks, may cause PARP-1 hyper-activation [37,38,39,40], leading to energy misbalances and ultimately resulting in a caspase-independent cell death [41]. Several modes of cell death are induced by high levels of cellular ROS, for example ferroptosis or parthanatos, and it remains to be established if zafirlukast is eliciting these types of cell death in ccRCC cells. Our observation that PARP-1 inhibition by Olaparib could partially rescue zafirlukast-induced cell death in 786-O cells might hint towards increased ROS production, oxidative damage, followed by PARP-1 hyper-activation and non-caspase cell death, but further experiments are required to confirm this hypothesis.

Zafirlukast-mediated cell death could be reduced by inhibiting HIF-2α in cells lacking a functional pVHL E3 ubiquitin ligase that thereby accumulate HIF-2α. As anticipated, inhibition of the proteasome and of HIF-2α degradation sensitizes wild-type pVHL-expressing cells towards zafirlukast-induced cell death. These findings suggest that increased or stabilized HIF-2α activity might be an important determinant for zafirlukast-mediated cell death. The contribution of additional HIF transcription factors to zafirlukast-induced cell death remains to be determined. In conclusion, our findings reveal important roles of ROS, pVHL and HIF-2α in zafirlukast-induced cell death that might lead to the development of novel therapeutic options in ccRCC.

## 4. Materials and Methods

### 4.1. Cell Culture and Chemicals

The *VHL*-mutated, human ccRCC cell line 786-O was obtained from Bernhard Brüne (Goethe University, Frankfurt am Main). The 786-O cells, stably transduced with empty vector or FLAG-tagged pVHL, were a kind gift from William Kaelin Jr. (Dana Farber Cancer Institute, Boston, MA, USA). Human embryonal kidney (HEK293T) and human colon carcinoma HT-29 cells were obtained from DSMZ (Braunschweig, Germany).

The 786-O cell lines were maintained in RPMI 1640 medium (ThermoFisher Scientific, Dreieich, Germany), containing 1% penicillin/streptomycin (P/S) (Invitrogen™, Waltham, MA, USA), 10% fetal calf serum (FCS) (Biochrom, Berlin, Germany) and 1% sodium pyruvate (ThermoFisher Scientific). The 293T cells were grown in Dulbecco’s modified Eagle’s medium (DMEM) GlutaMAX™-I medium (Life Technologies, Carlsbad, CA, USA), supplemented with 10% FCS and 1% P/S. HT-29 cells were cultivated in McCoy’s 5A Medium GlutaMAX™-I (Life Technologies, Inc., Eggenstein, Germany), supplemented with 1% P/S and 10% FCS. All cells were maintained at 37 °C in a humidified atmosphere with 5% CO_2_ and regularly monitored for mycoplasma infections.

Zafirlukast and the PARP-1 inhibitor Olaparib were obtained from Selleckchem (Houston, TX, USA). Human recombinant TNFα was obtained from (Biochrom, Ltd., Berlin, Germany), Nec-1s, GSK’872 and NSA were purchased from Merck (Darmstadt, Germany), carbobenzoxy-valyl-alanyl-aspartyl-[O-methyl]- fluoromethylketone (zVAD.fmk) was obtained from Bachem (Heidelberg, Germany), Enbrel (etanercept) from Pfizer (Berlin, Germany), erastin, α-Toc, NAC, BHA were obtained from Sigma-Aldrich. PT-2385 was purchased from BioVision (Milpitas, California, USA), MG-132 from Merck Millipore (Burlington, MA, USA) and VH298 was obtained from Axon Medchem (Groningen, The Netherlands).

### 4.2. Quantification of Cell Death

The indicated cell lines were seeded at appropriate densities for 24 h prior to stimulation in sterile 96-well plates (Greiner Bio-One, Kremsmünster, Austria). The degree of cell death was assessed by quantifying the fraction of PI (Sigma-Aldrich, P4864)-positive cells as a fraction of total cells, determined by Hoechst 33342 (Sigma-Aldrich, 14533) staining. Cell death imaging and quantification were performed using the ImageXpress Micro XLS Widefield High-Content Analysis System and MetaXpress Software according to the manufacturer’s instructions (Molecular Devices, Sunnyvale, CA, USA).

### 4.3. Western Blot Analysis and Antibodies

Cell lysates were generated by lysis of cells in lysis buffer (30 mM Tris HCl pH 7.4, 150 mM NaCl, 1% Triton-X100, 10% glycerol), supplemented with protease inhibitor cocktail (Roche, Grenzach, Germany), phosphatase inhibitors (1 mM sodium orthovanadate, 1 mM β-glycerophosphate, 5 mM sodium fluoride). Cells were lysed on ice for 30 min at 4 °C and centrifuged at 14,000 rpm for 25 min. Supernatants were collected and protein concentrations were determined and equalized using the BCA Protein Assay Kit (ThermoFisher Scientific). Samples were heated for 5 min at 95 °C in 2× Laemmli loading buffer (6× Laemmli: 360 nM Tris Base pH 6.8, 30% glycerol, 120 mg/mL SDS, 93 mg/mL DTT, 12 mg/mL bromophenol blue), followed by Western blot analysis.

Primary antibodies used in this study are: anti-VHL (clone S2647, #564183, BD Biosciences); anti-HIF-2α (#NB100-122, Novus Biologicals); anti-CysLTR1 (#AP01264PU-N, Origene); anti-TNFR1 (#3736S, Cell Signaling); anti-RIPK1 (#610459, BD Bioscience, Heidelberg, Germany); anti-MLKL (#14993S, Cell Signaling); anti-caspase-8 (#9746S, Cell Signaling); anti-RIPK3 (#13526S, Cell Signaling) and anti-vinculin (#V9131, Sigma/Merck). Appropriate horseradish peroxidase (HRP)-conjugated goat anti-mouse IgG and goat anti-rabbit IgG (Santa Cruz Biotechnology, Dallas, TX, USA) secondary antibodies were used and detected with enhanced chemiluminescence (Amersham Bioscience, Freiburg, Germany). Primary antibodies were used as 1:1000 in phosphate-buffered saline (PBS) supplemented with 0.2% Tween 20 (PBS-T) and 2% bovine serum albumin (BSA) and secondary antibodies were diluted 1:10,000 in PBS-T/5% non-fat milk powder. Representative blots of at least two independent experiments are shown.

### 4.4. Quantitative RT-PCR

To quantify gene expression the indicated cells were subjected to isolation of total RNA using the PeqGold RNA kit (Peqlab, Erlangen, Germany), according to the manufacturer’s instructions. Between 500–1000 ng of RNA per sample were used for cDNA synthesis using the RevertAID H Minus First Strand cDNA synthesis kit (Thermo Fisher Scientific). Quantification of gene expression was completed using SYBR green-based qRT-PCR employing the SYBR™ Green PCR Master Mix (Thermo Fisher Scientific) and the QuantStudio 7 Flex Real-Time PCR System (Applied Biosystems, Thermo Fisher Scientific. Relative RNA levels of the genes of interest were calculated relative to GAPDH as the housekeeping gene, using the 2^−ΔΔCT^-method [42]. At least three independent experiments in technical duplicates or triplicates were performed. All primers were purchased at Eurofins (Hamburg, Germany). Primer sequences are available upon request.

### 4.5. Statistical Analysis

Significance was assessed using Student’s *t*-test (two-tailed distribution, two-sample, equal variance) using Microsoft Excel, unless indicated otherwise. *p*-values < 0.05 are considered significant (* *p* < 0.05; ** *p* < 0.01; *** *p* < 0.001, n.s.: not significant).

## Data Availability

All relevant data are included in the manuscript.

## Supplementary Materials

The following supporting information can be downloaded at: https://www.mdpi.com/article/10.3390/ijms23073567/s1.
