# Supplementary material for: Zafirlukast Induces VHL- and HIF-2α-Dependent Oxidative Cell Death in 786-O Clear Cell Renal Carcinoma Cells
Source: Int J Mol Sci. 2022 Mar 25;23(7):3567. doi: 10.3390/ijms23073567 (PMC8999127; doi:10.3390/ijms23073567)
Supplement: Supplementary file 1 [file ijms-23-03567-s001.zip › ijms-1605549-supplementary.pdf]

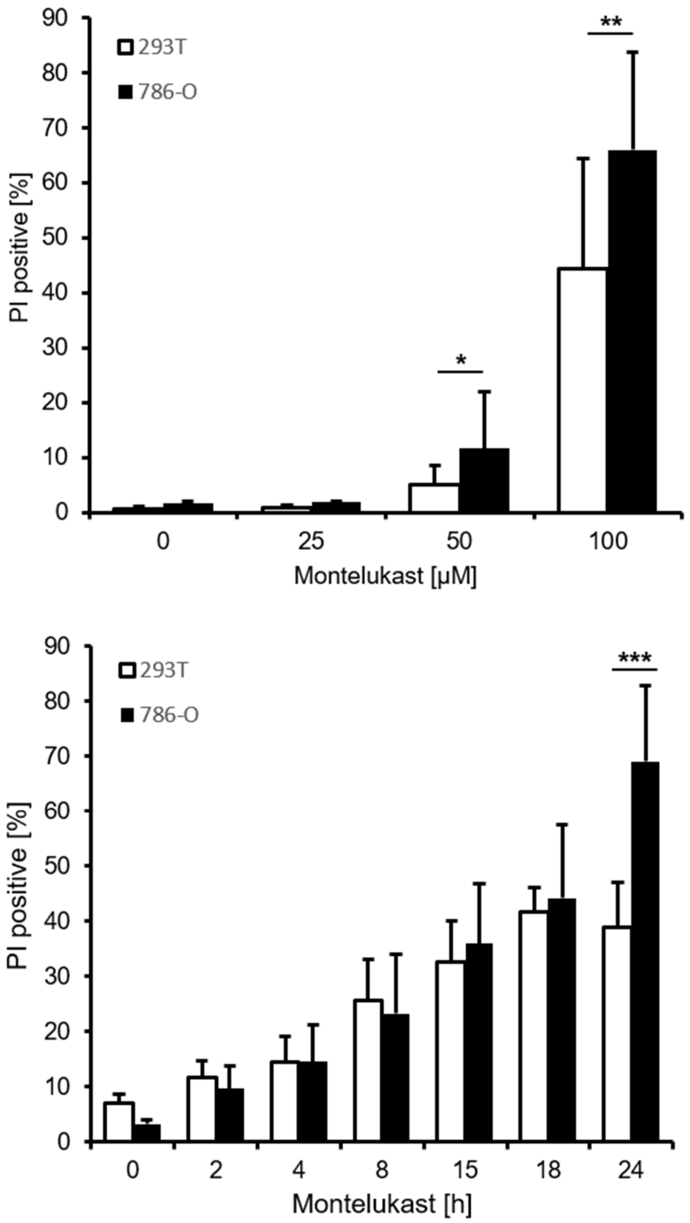

Figure S1. 786-O RCC cells are sensitive towards montelukast-induced cell death. 786-O and 293T cells were treated with the indicated concentrations of montelukast for 24 hours (*upper*) and indicated timepoints (100μM montelukast) (*lower*), after which cell death was determined by quantifying PI uptake. Data are presented as percentage of PI-positive cells and mean and SD of three independent experiments performed in triplicate are shown. * $P < 0.05$, ** $P < 0.01$, *** $P < 0.001$.

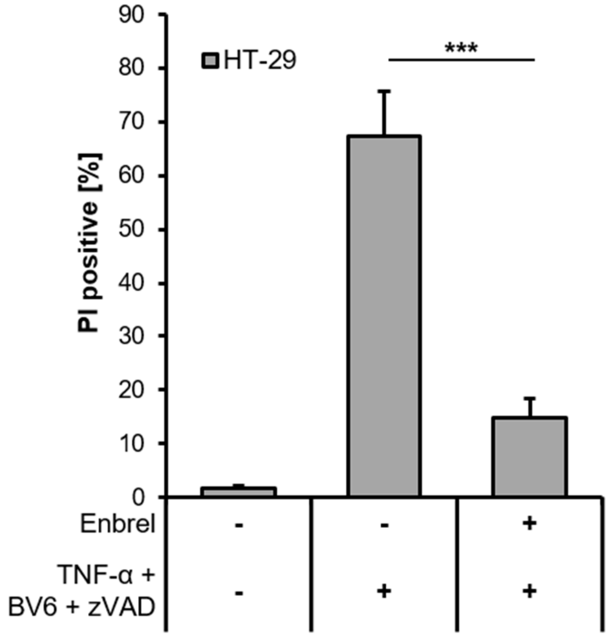

Figure S2. Enbrel-mediated inhibition of TNFα/BV6/zVAD.fmk-induced necroptosis in HT-29 cells. HT-29 colon carcinoma cells were pre-treated with 100 μg/ml of Etanercept (Enbrel) for 1 h, followed by treatment with 20 ng/ml TNFα, 5 μM BV6 and 20 μM zVAD.fmk for 24 h, after which cell death was determined by quantifying PI uptake. Data are presented as percentage of PI-positive cells and mean and SD of three independent experiments performed in triplicate are shown. *** P < 0.001.

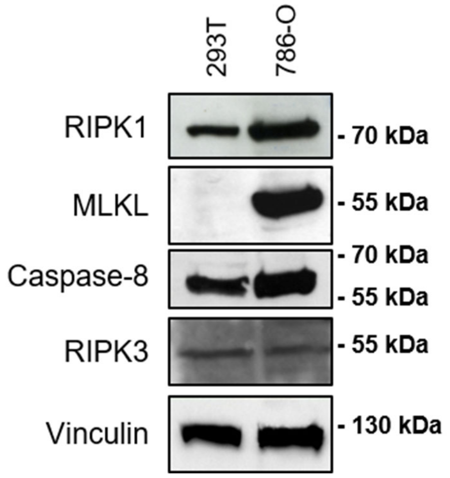

Figure S3. Expression of necroptotic key proteins in 293T and 786-O cells. Western blot analysis of RIPK1, RIPK3, caspase-8 and MLKL in 293T and 786-O cells. Vinculin served as loading control.

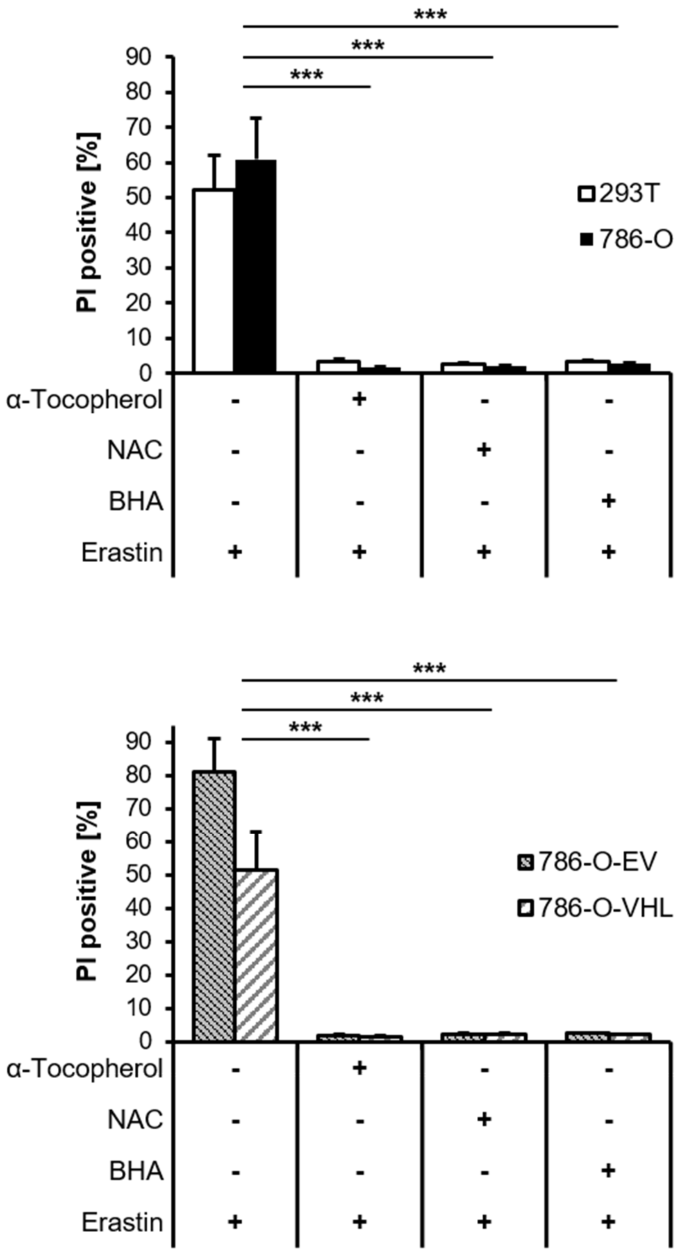

Figure S4. Antioxidants affect Erastin-induced cell death. 786-O and 293T (upper) and 786-O-EV and 786-O-VHL (lower) cells were pre-treated with α-Toc (100 μM), NAC (10 mM) and BHA (50 μM), followed by treatment with 10 μM Erastin for 24 h, after which cell death was determined by quantifying PI uptake. Data are presented as percentage of PI-positive cells and mean and SD of four independent experiments performed in triplicate are shown. *** P < 0.001.
